# Fiber-deprived diet weakens lung defense against antimicrobial-resistant *Klebsiella pneumoniae* and facilitates resistance phenotype in the gut microbiota

**DOI:** 10.1080/29933935.2026.2625617

**Published:** 2026-02-08

**Authors:** Mayra Fernanda Ricci, Clenio Silva Cruz, Viviani Mendes de Almeida, Mirna d' Auriol, Victor M. Rocha, Elayne C. Machado, Bruno Gallotti, Isabela Garbazza, Ana Maria Caetano Faria, Geovanni Dantas Cassali, Cristiana C. Garcia, Leiliane Coelho André, Flaviano S. Martins, Vinícius Abreu, Sintia Almeida, Angelica T. Vieira

**Affiliations:** a Laboratory of Microbiota and Immunomodulation, Department of Biochemistry and Immunology, Institute of Biological Sciences, Universidade Federal de Minas Gerais – UFMG, Belo Horizonte, MG, Brazil; b Laboratory of Toxicological Analysis (LATO), Department of Clinical and Toxicological Analysis, Faculty of Pharmacy, Universidade Federal de Minas Gerais – UFMG, Belo Horizonte, MG, Brazil; c Laboratory of Immunobiology, Department of Biochemistry and Immunology, Institute of Biological Sciences, Universidade Federal de Minas Gerais – UFMG, Belo Horizonte, MG, Brazil; d Laboratory of Comparative Pathology, Department of General Pathology, Universidade Federal de Minas Gerais – UFMG, Belo Horizonte, MG, Brazil; e Laboratório de Vírus Respiratórios, Exantemáticos, Enterovírus e Emergências Virais, Instituto Oswaldo Cruz, Fiocruz, Rio de Janeiro, RJ, Brazil; f Grupo Integrado de Pesquisas em Biomarcadores, Instituto René Rachou, Fiocruz, Belo Horizonte, MG, Brazil; g Laboratory of Biotherapeutic Agents, Department of Microbiology, Institute of Biological Sciences, Universidade Federal de Minas Gerais-UFMG, Belo Horizonte, MG, Brazil; h Instituto de Ciências Exatas e Naturais – ICEN – Universidade Federal do Pará – UFPA, Pará, PA, Brazil; i Instituto Evandro Chagas - IEC, Setor de Tecnologia da Informação e Comunicação – SOTIC, Pará, PA, Brazil; j University of Strasbourg Institute for Advanced Study (USIAS) Fellow, Institute for Translational Medicine and Liver disease – ITM (INSERM Unit -U1110), University of Strasbourg, Strasbourg, France

**Keywords:** Microbiota, inflammation, antimicrobial-resistance, Gut–Lung-axis, resistome

## Abstract

The rapid spread of antimicrobial-resistant (AMR) bacteria is a major global health challenge. The misuse of antibiotics and infections by resistant pathogens drive the dissemination of resistance genes, the human microbiota of which serve as reservoirs. Disruptions in the host–microbiota balance, which are influenced by diet, can increase resistance genes. Low-fiber diets are linked to gut dysbiosis, infection susceptibility, and weakened defenses. Here, we report that dietary fiber deprivation induced significant alterations in the gut microbiota of C57BL/6 mice, leading to reduced host tolerance to lung infection by the AMR strain *Klebsiella pneumoniae* B31 (KP 31) with higher levels of ampicillin-resistant *Enterobacteriaceae* and marked shifts in the gut microbial composition. Germ-free mice that received fecal transplants from fiber-deprived donors also displayed exacerbated inflammatory pathology following KP 31 infection. Infection further increased the abundance of cultivable resistant *Enterobacteriaceae* in the gut and was associated with the modulation of short-chain fatty acid (SCFA) levels, particularly propionate. Propionate appears to support antimicrobial activity, and its decrease *in vitro* promotes bacterial growth. Our findings highlight that the gut microbiota is a crucial reservoir for resistance genes. Low-fiber diets impair lung defenses and promote resistome expansion after AMR infection. Understanding these dynamics and their influencing factors is essential for strategies to combat antimicrobial resistance (AMR).

## Introduction

The widespread emergence of antimicrobial-resistant (AMR) bacteria in the 21st century is alarming, and the recent COVID-19 pandemic has further accelerated the spread of AMR.^1^^,^^2^ Existing countermeasures against AMR have not achieved the expected containment outcomes, suggesting a lack of comprehensive understanding of other contributing factors to the rise in AMR. While the misuse and overuse of antimicrobials remain the primary drivers of AMR, uncontrolled infections caused by AMR pathogens also contribute significantly to the dissemination of resistant microorganisms among individuals, creating new hotspots of AMR genes.^3^^,^^4^ The human microbiota, composed of trillions of microorganisms (mostly bacteria), forms a complex and well-recognized ecosystem^5-7^ and can act as reservoirs for resistance genes.

Host–microbiota composition is increasingly recognized as a critical determinant of health and disease, with environmental factors—particularly diet—playing a central role in modulating this delicate balance. Poorly balanced diets are associated with gut microbiota dysbiosis, impaired immune defenses and higher susceptibility to pathogenic infections.^8-15^ Dysbiosis also promotes the expansion of bacteria carrying AMR genes and contributes to the development of inflammatory diseases.^16^

For instance, the nutritional changes observed in Western societies over recent decades have been correlated with an increase in the prevalence of inflammatory conditions.^14^^,^^17^ Emerging evidence suggests that diet-induced dysbiosis in the gut microbiota may drive inflammatory and infectious diseases. Additionally, higher rates of horizontal gene transfer in the microbiomes of individuals in industrialized and urban populations suggest that lifestyle factors significantly influence the microbiome resistome.^18^^,^^19^ Conversely, balanced diets—particularly those rich in fiber—are strongly correlated with host homeostasis and lower rates of infectious diseases.^9^^,^^10^^,^^17^^,^^20^ Such diets can promote beneficial microorganisms over pathogens and pathobionts, including members of the ESKAPE group (*Enterococcus faecium*, *Staphylococcus aureus*, *Klebsiella pneumoniae*, *Acinetobacter baumannii*, *Pseudomonas aeruginosa*, and *Enterobacter* spp.).^16^^,^^21^^,^^22^ Many *Enterobacteriaceae* strains, including *K. pneumoniae*, exhibit resistance to nearly all available antibiotics.^1^^,^^23^ Although *K. pneumoniae* is a major global source of antibiotic resistance; it is also an essential component of the microbiota and typically exists as a nonharmful symbiont under normal conditions. *K. pneumoniae*, a Gram-negative pathobiont commonly found in the gut, is a major cause of both hospital- and community-acquired pneumonia and is associated with severe respiratory failure due to excessive inflammation. It also causes a range of systemic infections, including sepsis, meningitis, liver abscesses, and ICUs in both healthy and immunocompromised individuals.^24^ Its high pathogenicity stems from virulence factors such as a protective capsule, lipopolysaccharides, adhesins, and iron acquisition systems.^25^ Compounding this threat is its increasing AMR, particularly in multidrug-resistant (MDR) strains, which the WHO has designated as a critical priority for new therapies.^5^^,^^26^ For example, the antimicrobial therapy in hospitalized patients promotes gut microbiota alteration and enhances the susceptibility to pulmonary infection. It is linked via SCFA to weakened lung immunity against *K. pneumoniae.*^27^ This is especially concerning in developing countries, where mortality rates from resistant infections are disproportionately high. In these regions, the increasing adoption of Western diets—high in refined sugars and fats but low in fiber—further exacerbates vulnerability to respiratory infections.^28^ Low-fiber diets are known to disrupt the gut microbiota composition, impair intestinal barrier function, and weaken immune responses, all of which can facilitate systemic infections and bacterial translocation.^29^^,^^30^

In this study, we investigated how the deprivation of fiber in the diet affects the susceptibility of the host to lung infections caused by AMR *K. pneumoniae*. To address this, we used a mouse model fed with a low-fiber diet and infected with an AMR pathogenic strain of KP B31. We also performed fecal microbiota transplantation from these mice to germ-free mice as a proof-of-concept approach. This allowed us to demonstrate that alterations in the gut microbiota contribute to this phenotype. Our findings highlight the critical role of the intestinal microbiota as a reservoir of resistance genes, emphasizing that nutritional imbalances can facilitate the expansion of the resistome in hosts following infection by AMR bacteria.

## Methods

### Laboratory animals

Female C57BL/6J mice, approximately 8 weeks of age, were obtained from the animal facility of the Federal University of Minas Gerais (UFMG), Minas Gerais, Brazil. The animals were housed in plastic cages under standard conditions (26 °C, 12-h light/dark cycle) and provided with steam-sterilized food (Nuvilab®, Nuvital, Brazil) and water *ad libitum*. Additionally, approximately 8-week-old female Germ-Free Swiss/NIH mice were used. These mice were derived from a germ-free nucleus (Taconic Farms, Germantown, USA) and maintained in sterilized flexible plastic isolators (Standard Safety Equipment Co., Palatine, USA) using classical gnotobiology techniques at the Gnotobiology Laboratory of UFMG. All animal procedures were conducted following the guidelines of the Guide for the Care and Use of Laboratory Animals of the Brazilian National Council for Animal Experimentation (http://www.cobea.org.br/) and Brazilian Federal Law 11.794 (8 October 2008). The study protocol was reviewed and approved by the Institutional Animal Ethics Committee of UFMG (protocol nº CEUA/UFMG 281/2020).

### Experimental design

We conducted two independent experiments using C57BL/6J mice, randomly assigned to one of two dietary groups: (1) the control AIN-93 diet or (2) the AIN-93-modified low-fiber (LF) diet. In both experiments, the mice were fed their respective diets for 21 d, followed by intratracheal infection. In the first experiment, the mice were euthanized 48 h postinfection (hpi). In the second experiment, euthanasia occurred 15 d postinfection (dpi). Each experiment was conducted twice, with a minimum of four mice analyzed per group and time point.

### Diet

The control group was fed the standard AIN-93 diet, with no modifications in fiber content.^3^ In contrast, the low-fiber (LF) group received a modified AIN-93 diet, in which all cellulose was replaced with sucrose, without the addition of any other fiber sources. The composition and percentages of each component in the control and LF diets are detailed in the supplementary material (Table S1).

### *K. pneumoniae* intratracheal infection

The bacterium used in the intratracheal pulmonary infection experiments was *K. pneumoniae B31* (KP B31), a clinical isolate with a multidrug-resistance profile.^15^ For the procedure, the animals were anesthetized intraperitoneally (i.p.) with 80 mg/kg ketamine and 15 mg/kg xylazine i.p., and then their trachea was exposed through a skin incision, and 25 μL of the suspension containing 1 × 10^6^ CFU/mL of KP B31 or sterile saline for mock controls was injected with a 26-gauge needle.^17^ After infection, the skin was sutured, and the mice were monitored and then euthanized as described in the experimental design.

### Fecal microbiota transplantation (FMT) to GF mice

Fecal samples of each C57BL/6J mice from control and low-fiber diets were weighed and resuspended in 0.9% sterile saline (NaCl) solution (100 mg/mL). The preparation was conducted in an anaerobic chamber. The fecal solution was maintained at 4 °C for 10 min to precipitate insoluble fecal components, and a 100 μL of aliquot was used for the oral gavage of germ-free Swiss/NIH mice. Following FMT, the germ-free (GF) mice were maintained for 14 d to allow stabilization of the newly acquired microbiota. After this period, the mice were infected intratracheally with *K. pneumoniae* B31 and euthanized 48 h postinfection. The Swiss/NIH germ-free mice received a standard, simplified diet and sterile, filtered water throughout the experiment, including after fecal transfer. After the microbiota stabilization period, the Swiss/NIH GF mice were infected and monitored for 48 h before euthanasia.

### Bronchoalveolar lavage collection and analysis

Following euthanasia, bronchoalveolar lavage (BAL) fluid was obtained by inserting 1 mL of phosphate-buffered saline (PBS) into the lungs through a 20-gauge catheter attached to a 1 mL syringe. Then, the aliquot was collected, centrifuged, and resuspended in 100 µL of sterile saline, and the total leukocytes were quantified via a Neubauer counting chamber.^17^ KP B31 was quantified by plating BAL samples directly on MacConkey agar (Sigma, Germany) and incubated under aerobic conditions for 24 h at 37 °C. After incubation, the colonies were counted, and the data were expressed as the log_10_ of CFU per mL of BAL.

### Cultivable fecal *Enterobacteriaceae* identification and antimicrobial resistance test

After the stabilization period of the microbiota following FMT, fecal samples from control and LF groups were collected. The samples were plated on MacConkey agar (Sigma, Germany) and incubated aerobically at 37 °C for 24 h. Colony-forming units (CFU) were enumerated and expressed as log_10_ CFU per mg on feces. *Enterobacteriaceae* colonies displaying distinct morphologies were selected, purified, and subjected to antimicrobial susceptibility testing (AST). Strain identification was carried out by matrix-assisted laser desorption/ionization–time of flight (MALDI-TOF) mass spectrometry using the FlexControl MicroFlex LT system (Bruker Daltonics), as previously described.^31^ Before identification, calibration was performed with the bacteria *Escherichia coli* DH5α test standard (Bruker Daltonics).

### Myeloperoxidase assays (MPO)

Lung tissue was homogenized in 1.9 mL of PBS and centrifuged at 12,000 g for 10 min at 4 °C. The samples were then resuspended in hexadecyltrimethylammonium buffer (HTAB, 0.5% w/v) and subjected to 3 cycles of freezing in liquid nitrogen. After these cycles, the samples were centrifuged, and the supernatant was collected for the enzymatic assay. For the MPO enzymatic assay, the substrate 3,3ʹ,5,5ʹ-tetramethylbenzidine (TMB, Sigma, USA) and H_2_O_2_ in Tris-hydrochloric acid (HCl) buffer was used, and the reaction was stopped with 1 M H_2_SO_4_. MPO activity was calculated from the optical density at 450 nm using a microplate spectrophotometer (Epoch, BioTek Instruments, USA).

### Short-chain fatty acids (SCFAs) measurement

The SCFA measurements of the feces were performed as previously described.^1^ Seven-point external calibration curves were adopted to quantify the fecal samples using analytical-grade SCFA (Sigma, Germany) as standards.

### Total gastrointestinal (GI) transit assay

The evaluation of intestinal physiology was performed as previously described.^32^ The total GI transit time (total transit time) represents the interval between time 0 and the time of the first observance of carmine red dye in the stool.

### Histopathology analysis

Formalin-fixed lungs were dehydrated gradually in ethanol, embedded in paraffin, cut into 4-µm sections, stained with H&E and examined under light microscopy and scored by a pathologist blinded to the experiment. The inflammatory score of 13 points was determined with hematoxylin and eosin (H&E) stained slides and airway, vascular, and parenchymal inflammation (0, absent; 1, minimal; 2, slight; 3, moderate; 4, marked; and 5, severe) were evaluated.^33^^,^^34^

### Fecal DNA extraction and sequencing

Fecal samples were stored in a freezer at −70 °C. DNA was isolated using an AllPrep PowerFecal Pro DNA/RNA Kit (Qiagen, USA). For shotgun sequencing, Illumina libraries were prepared using a TruSeq Nano DNA Library Prep Kit (Illumina, USA) according to the manufacturer's protocol and sequenced using the HiSeq 4000 sequencing platform (Illumina, USA) with a read length of 100 bp (paired) for all samples.

### Sequence data processing and inferring gut microbiota composition

Metagenomic reads were quality filtered and trimmed using Sickle v1.33.^35^ High-quality reads were assembled with MetaSPAdes v3.15.0.^36^ Given the inherent complexity of shotgun metagenomic data and the pronounced strain-level variability characteristic of gut microbial communities, an initial grouping of samples was applied prior to downstream analyses,^37^^,^^38^ following established metagenomic assembly strategies that improve coverage and genome reconstruction while reducing computational bias.

The samples were organized according to experimental conditions, defined by the combination of diet and infection status: (1) standard diet + *K. pneumoniae* B31 infection (SDDN1–SDDN3); (2) low-fiber diet + infection (SDDN4–SDDN6); (3) standard diet, noninfected (SDDN7–SDDN10); and (4) low-fiber diet, noninfected (SDDN11–SDDN14). This grouping reflects the biological experimental unit of the study, which is consistent with controlled murine models conducted under cohousing conditions. In such settings, a shared environment, coprophagy, and continuous microbial exchange substantially reduce within-group independence, rendering group-level inference biologically more appropriate than individual-level comparisons. Accordingly, all inferential analyses were designed to compare predefined treatment groups (diet × infection) rather than individual libraries, avoiding misinterpretation of sample-level variability and aligning with best practices for microbiome studies in animal models.^37^^,^^39^ From a technical perspective, metagenomic analyses face challenges in resolving strain-level diversity owing to sequencing depth heterogeneity and computational constraints. Preanalysis stratification using experimental metadata mitigates biases associated with assembly fragmentation, low-abundance taxa, and uneven coverage, thereby enhancing the robustness and interpretability of downstream taxonomic, functional, and resistome profiles.^40^^,^^41^

### Taxonomic, functional, and resistance profiling of the gut microbiome

Taxonomic classification of metagenomic reads was performed using Kraken2 with a reference microbial database, followed by postprocessing with TaxProfile to aggregate reads and estimate phylum-level abundances.^42^^,^^43^ Community composition and ecological metrics were further analyzed using the microeco R package.^44^ Alpha diversity was assessed using Shannon's index and observed richness, while beta diversity was calculated via Bray–Curtis dissimilarity and visualized through principal coordinates analysis (PCoA). All diversity analyses were conducted using the R packages *phyloseq,*^45^ *vegan*^46^ *ggplot2*, *ggpubr*, and *pairwiseAdonis.*^47^ Group differences in diversity metrics were evaluated using Kruskal–Wallis tests, followed by Dunn's post hoc tests with Benjamini‒Hochberg correction for multiple comparisons. Additionally, rarefaction curves based on Hill numbers (*q* = 0) were constructed with the *iNEXT* package to estimate species richness under varying sequencing depths, allowing interpolation and extrapolation from individual-based data.^48^

Antibiotic resistance genes (ARGs) were identified and annotated using the FuncScan pipeline, incorporating the CARD database.^49^ ARG abundances were calculated both globally and by specific taxa (e.g., *Pseudomonadota*), using normalized counts. Co-occurrence patterns among ARG classes were explored via pairwise Spearman correlation, and relationships were visualized using heatmaps. The functional genes from the metagenomic reads were annotated into metabolic pathways, and the functional metabolic profiles were compared across groups using hierarchical clustering based on Euclidean distances and Ward's linkage method. Heatmaps and cluster diagrams were created using Python 3 packages including *NumPy*, *pandas*, *seaborn*, and *matplotlib.*^50^

### *In vitro* assays

*K. pneumoniae* B31 was cultured in brain heart infusion (BHI) (Sigma-Aldrich, USA) at 37 °C for 24 h. After the incubation period, the cultures were centrifuged and diluted to the final concentration of 1 × 10⁹ CFU/ml in Luria–Bertani (LB) (Sigma-Aldrich, USA) minimal medium supplemented with acetate at concentrations of 30, 60, and 90 mM; propionate at concentrations of 10, 20, and 30 mM; and butyrate at concentrations of 10, 20, and 30 mM were used as previously described.^51^ Subsequently, 200 µL of the bacterial suspension of *K. pneumoniae* B31 prepared in LB medium with the indicated concentrations of each SCFA was added to 96 wells of microplates. The microplates were then incubated at 37 °C, and the optical density (OD) was measured every 2 h using a microplate reader at a wavelength of 450 nm. For the controls, the medium was prepared without the addition of the SCFAs tested in the experiments.

### Statistical analysis

Statistical analyses and graphs were done using GraphPad Prism10 (GraphPad Software, La Jolla, CA, United States). The data are shown as mean ± standard deviation (SD). Data that presented normal distributions (*p* > 0.05 by Shapiro–Wilk tests) were evaluated by Student's *t*-test, one-way and two-way analysis of variance (ANOVA), depending on the experimental design. Following significant ANOVAs, we performed a post hoc test according to the coefficient of variation (CV): Tukey (CV ≤ 15%), Student's Newman–Keuls (CV entre 15%–30%) and Duncan (CV > 30%). The differences were considered significant when *p* < 0.05. For all comparative analyses of the metagenome data, statistical tests were conducted using R (v4.3.0) or Python 3.11. One-way ANOVA followed by Tukey's HSD test was used to assess group-level differences in functional and ARG profiles. Categorical comparisons (e.g., ARG class frequencies) were performed using chi-square tests. Differences in diversity metrics (alpha and beta diversity) were evaluated as described above. Fold changes in metabolite-related abundance were calculated relative to the Std-NI group. A *p*-value < 0.05 was considered statistically significant unless otherwise stated.

## Results

### Low-fiber diets promote body weight loss, enhances myeloperoxidase activity and amplifies gut microbiota changes that exacerbate lung damage during *K. pneumoniae* B31 infection

**Figure 1.**
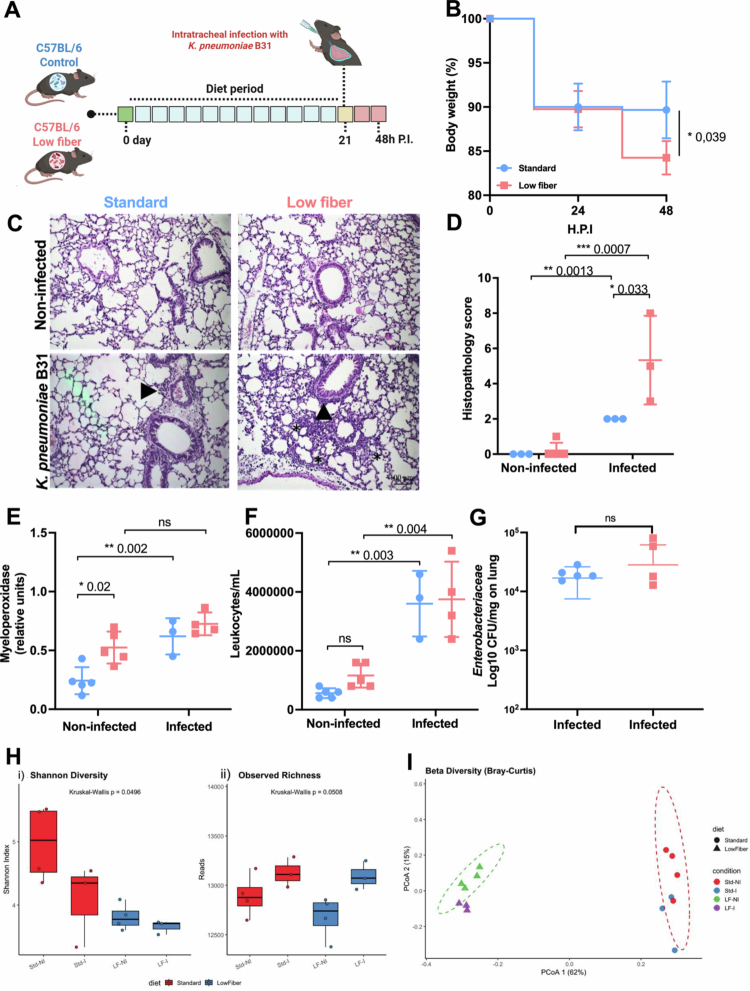
A low-fiber diet reduced the tolerance of mice to pulmonary infection by antimicrobial-resistant *Klebsiella pneumoniae* (KP B31). (A) C57BL/6 mice were fed a standard diet or a low-fiber diet for 21 d, followed by intratracheal infection with KP B31. The mice were monitored for 48 h postinfection to assess morbidity and mortality. (B) Weight change in C57BL/6 mice over 48 h postinfection. (C) Representative photomicrographs of lung tissue from uninfected and KP B31-infected C57BL/6 mice. (D) Histopathological scores of the lung parenchyma of uninfected and KP B31-infected C57BL/6 mice, with a total of 13 points. (E) Quantification of myeloperoxidase (MPO) levels in lung tissue from uninfected and KP B31-infected C57BL/6 mice. (F) Leukocyte counts in bronchoalveolar lavage (BAL) fluid from uninfected and KP B31-infected C57BL/6 mice. (G) Quantification of *Enterobacteriaceae* isolated from the lung tissue of KP B31-infected C57BL/6 mice. Statistical comparisons between the standard diet and low-fiber diet groups means were performed using unpaired Student's t test. Significance levels: ns = *p* > 0.05; **p < 0.01; ****p < 0.0001. Images were acquired using a 20x objective. The arrows in the photomicrographs indicate inflammatory infiltrates. The asterisk indicates parenchymal inflammation. *N* = 3–5 mice per group (variation due to technical loss during tissue processing). (H) Alpha diversity based on taxonomic profiles derived from shotgun metagenomic data across diet and infection conditions. (1) The Shannon diversity index and (2) observed richness (total number of classified reads) were computed using the phyloseq framework. Boxplots show the distribution of alpha diversity per group, with individual samples displayed as jittered points. The colors indicate the following dietary categories: standard (red) and low-fiber (blue). Statistical differences among groups were assessed using Kruskal–Wallis tests (Shannon: *p* = 0.0496; Observed: *p* = 0.0508), followed by Dunn's post hoc tests with Benjamini‒Hochberg correction. Significant pairwise differences (adjusted *p* < 0.05) are reported in Supplementary Table S2. (I) Beta diversity of gut microbial communities based on shotgun metagenomic data. Principal coordinates analysis (PCoA) based on Bray‒Curtis dissimilarity, illustrating the compositional differences in the gut microbiota across the experimental groups. Each point represents a sample, colored by condition (Std-NI: standard diet, no infection *N* = 4; Std-I: standard diet, infection *N* = 3; LF-NI: lover fiber diet, no infection *N* = 4; LF-I: lower-fiber diet, infection *N* = 3) and shaped by diet (standard: circles; low-fiber: triangles). The first two axes explain 62% and 15% of the total variation, respectively. PERMANOVA tests were performed and are reported in Supplementary Table S3. Ellipses denote 95% confidence intervals for each group, highlighting distinct microbial community structures associated with dietary fiber content and infection status.

To evaluate the impact of a low-fiber diet on host tolerance to pulmonary infection induced by *K. pneumoniae* B31, C57BL/6 mice were fed for 21 d with one of two diets: (1) a standard diet (Std-C), containing a standardized amount of fiber (2%–5%), or (2) a low-fiber (0%) diet (LF-C) (Supplemental information Figure S1A,B). After 21 d on the respective diets, no differences in body weight or total transit time were observed between the standard and low-fiber diet groups (Supplemental information Figure S1C,D). Following the diet period, the mice were intratracheally infected with *K. pneumoniae* B31, as outlined in the experimental design (Figure 1A). No mortality was observed in either the standard or low-fiber diet groups. However, mice on the low-fiber diet exhibited a greater percentage of body weight loss compared to those on the standard diet at 48 h after infection (Figure 1B). Histopathological analysis of the lungs revealed increased inflammatory infiltration in infected mice from the standard and low-fiber diets Compared to their respective non-infected groups. Also, in the KP B31 infection, we observed an increase in the inflammatory score in the low-fiber diet compared to the standard diet. (Figure 1C,D). We observed an increase in myeloperoxidase (MPO) levels, an indirect marker for neutrophil quantification, in the lungs of noninfected mice in low-fiber diet compared to standard diet. Additionally, we observed an increase in MPO in the standard group infected compared to the noninfected standard diet group. Although myeloperoxidase activity was elevated in both infected groups (standard diet and low-fiber diet) compared to the noninfected (control) groups, the infection did not further increase the levels in the low-fiber diet group compared with both infected groups (Figure 1E). The leukocyte count in the lungs was greater in the infected standard and low-fiber diet groups compared to their respective noninfected counterparts (Figure 1F). No differences in the quantification of bacterial colony-forming units (CFU) of cultivable *Enterobacteriaceae* were observed in the lungs between the infected groups (Figure 1G).

Considering the lung‒gut axis, we performed metagenomic analyses to compare the gut microbiota modulation between those mice exposed to different diets and to characterize the microbiota changes induced by KP B31 lung infection (Figure 1H). Our results revealed that dietary fiber content had a profound effect on gut microbial diversity and composition. Mice maintained on a standard diet exhibited significantly higher Shannon diversity and observed richness compared to those fed a low-fiber diet (Figure 1H) (Supplemental information Table S2). Furthermore, infection with KP B31 led to a moderate reduction in microbial diversity, particularly within the standard diet group, suggesting an additive effect of pulmonary infection on gut dysbiosis. Beta diversity analysis based on Bray–Curtis dissimilarity demonstrated distinct clustering of microbiota profiles, primarily according to dietary intervention, rather than infection alone (Figure 1I) (Supplemental information Table S3). The separation along the principal coordinates indicated that a low-fiber diet consistently shifted the microbial community structure, supporting the hypothesis that the gut environment is predominantly shaped by nutritional inputs, with systemic infections exerting secondary but detectable impacts. These findings reinforce the integrative nature of the lung–gut axis, where dietary modulation and respiratory infections converge to influence gut microbial ecology.

### Low-fiber intake drives gut microbiota dysbiosis and resistance expansion in *Enterobacteriaceae* following infection

**Figure 2.**
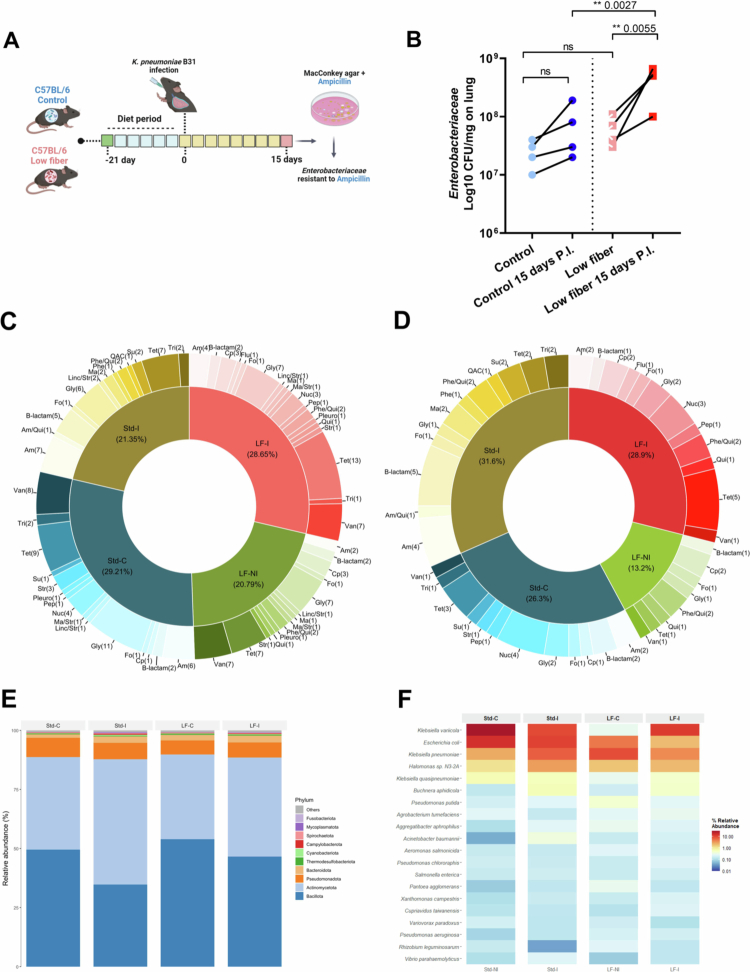
A low-fiber diet increases the amount of ampicillin-resistant *Enterobacteriaceae* following pulmonary infection with KP B31. (A) C57BL/6 mice were fed a standard diet or a low-fiber diet for 21 d, followed by intratracheal infection with KP B31, and monitored for up to 15 d postinfection. (B) Quantification of ampicillin-resistant *Enterobacteriaceae* isolated from C57BL/6 mice's feces before infection and 15 d after intratracheal infection with KP B31. *N* = 4. Characterization of the antibiotics identified in the metagenome of the phylum (C) Total and (D) *Pseudomonadota*. (E) Top 10 relative abundance in the phylum. (F) Top 20 species of *Pseudomonadota.* Statistical comparisons between the standard diet and low-fiber diet groups means were performed using paired Student's t test. Significance levels: ns = *p* > 0.05; **p < 0.01. STD (standard diet, no infection, *N* = 4), STD-I (standard diet, with infection, *N* = 3), LF (lower fiber, no infection, *N* = 4), and LF-I (lower fiber, with infection, *N* = 3).

To evaluate the impact of a low-fiber diet on colonization by ampicillin-resistant *Enterobacteriaceae*, we intratracheally infected mice fed either a standard or low-fiber diet with KP B31. On the day of infection and 15 d postinfection, we quantified ampicillin-resistant *Enterobacteriaceae* in the feces of both groups (Figure 2A). In the standard diet group, there was no statistically significant change in the level of ampicillin-resistant *Enterobacteriaceae* following infection. No differences were observed for both the control and low-fiber diets. In contrast, mice under low-fiber diet showed a significant increase in ampicillin-resistant *Enterobacteriaceae* 15 d after infection, with levels significantly higher than those observed in the standard diet group (Figure 2B). Because a time-matched noninfected low-fiber cohort was not analyzed at day 15, we cannot exclude diet-driven temporal effects independent of infection.

The analysis of the overall gut microbiota composition across antibiotic resistance classes showed no significant differences among the groups (Figure 2C). However, when we focused specifically on the *Pseudomonadota* phylum, we observed a broader antibiotic resistance profile in the low-fiber diet group (34.8%) compared to the other groups (Figure 2D). At the family level, community composition analysis revealed an increased relative abundance of *Actinomycetota* in the standard diet group after infection compared to uninfected controls (Figure 2E, light blue). In the low-fiber group, an increase in *Bacillota* abundance was observed in the control diet group (Figure 2E, dark blue). Finally, species-level analysis showed a marked increase in *K. pneumoniae* in the low-fiber diet group compared to both the uninfected controls and the infected mice on the standard diet (Figure 2F).

### Germ-free mice receiving FMT from low-fiber-fed donors mimic exacerbated inflammatory pathology associated with *K. pneumoniae* infection

**Figure 3.**
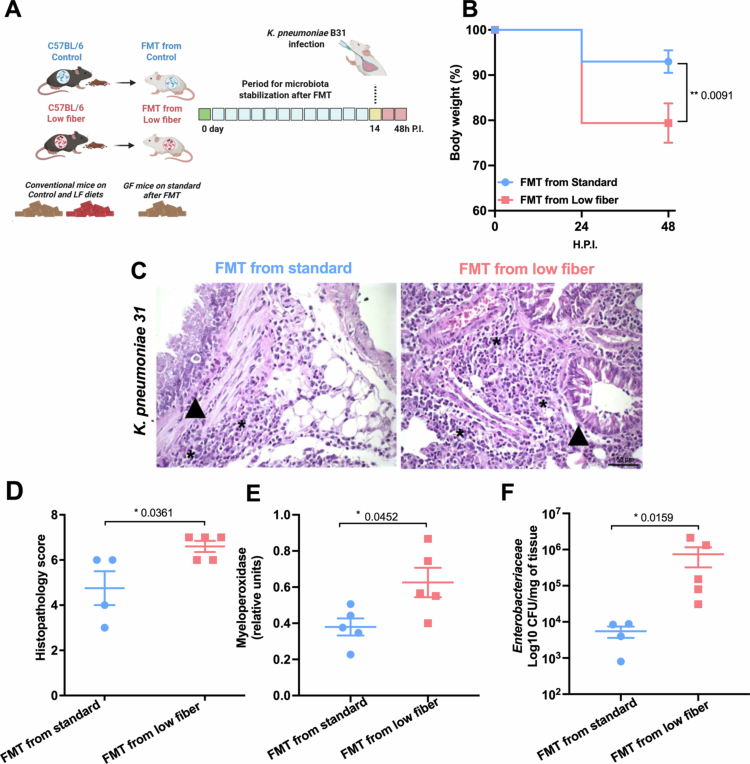
Fecal microbiota transfer (FMT) to germ-free mice recapitulates exacerbated inflammatory pathology following pulmonary, with KP B31 observed in conventional C57BL/6 mice fed with low-fiber diet. (A) Feces were collected from C57BL/6 mice fed with a standard diet or a low-fiber diet for 21 d and transferred to germ-free (GF) mice. After a microbiota stabilization period, the GF mice were allocated intoto the FMT from standard and FMT from low-fiber groups. The mice were subsequently infected intratracheally with KP B31 and monitored for 48 h. (B) Weight change in mice from the FMT from standard and FMT from low-fiber groups over 48 h postinfection. (C) Representative photomicrographs of lung tissue from FMT from standard and FMT from low-fiber mice infected with KP B31. (D) Histopathological score of the lung parenchyma in FMT from standard and FMT from low-fiber mice infected with KP B31. Images were acquired using a 40x objective. The arrows in the photomicrographs indicate inflammatory infiltrates. The asterisk indicates parenchymal inflammation. (E) Quantification of myeloperoxidase (MPO) levels in the lung tissue of FMT from standard and FMT from low-fiber mice infected with KP B31. (F) Quantification of *Enterobacteriaceae* in lung tissue of FMT from standard and FMT from low-fiber mice infected with KP B31. *N *= 4–5 mice per group (variation due to technical loss during tissue processing). Blue: GF mice colonized with microbiota from standard diet-fed mice (FMT from standard). Red: GF mice colonized with microbiota from low-fiber diet-fed mice (FMT from low-fiber). After FMT, the GF mice were fed with commercial sterile chow diet. Statistical comparisons between group means were performed using unpaired Student's *t*-test. Significance levels: ns = *p* > 0.05; *p < 0.05.

In order to determine whether the gut microbiota structure of conventional mice fed a low-fiber diet mediates altered host responses to infection with the antimicrobial-resistant KP B31 strain, we performed fecal microbiota transplantation (FMT) in germ-free mice (Figure 3A). The GF mice were allocated to the FMT standard and FMT low-fiber groups, and after the microbiota stabilization period, they were infected intratracheally with KP B31. After 48 h of infection, GF mice from FMT low-fiber group demonstrated greater weight loss when compared with GF mice from FMT standard group (Figure 3B). After infection, histopathological analysis of the lungs of the FMT low-fiber diet group showed an increased inflammatory tissue infiltration in the peribronchiolar regions (arrow) and lung parenchyma (asterisk) compared with mice from the FMT standard diet group (Figure 3C). Following these data, we observed an increased score of lung parenchymal inflammation in the FMT low-fiber diet group compared with the FMT standard diet group (Figure 3D). Furthermore, we observed a significant increase in myeloperoxidase levels (Figure 3E) in the lung tissue of the mice from the FMT low-fiber diet group compared with those in the FMT standard diet group. Additionally, we observed an increase of *Enterobacteriaceae* CFU in the lung tissue of FMT low-fiber diet mice compared to FMT standard diet mice (Figure 3F). These data suggest that the microbiota is related to the phenotype of increase pulmonary pathology following infection with KP B31 in mice.

### Dietary fiber deprivation limits cultivable *Enterobacteriaceae* diversity

**Figure 4.**
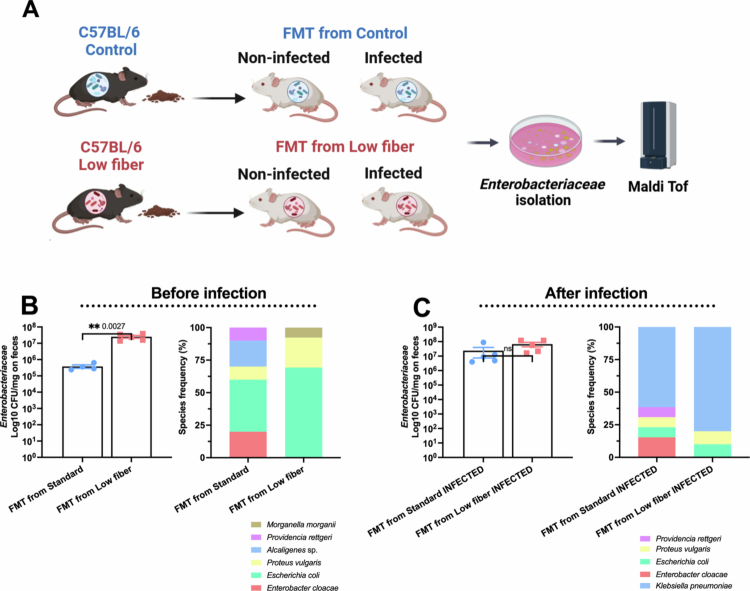
FMT reduces the diversity of *Enterobacteriaceae* species in GF mice. (A) Following FMT from C57BL/6 mice fed with standard diet or a low-fiber diet to GF mice, *Enterobacteriaceae* strains were isolated from the FMT from standard and FMT from low-fiber groups, both infected and uninfected, and identified using MALDI-TOF. (B) Quantification and relative frequency of *Enterobacteriaceae* species isolated from GF mice in the FMT from standard and FMT from low-fiber groups prior to pulmonary infection with KP B31. (C) Quantification and relative frequency of *Enterobacteriaceae* species isolated from GF mice in the FMT from standard and FMT from low-fiber groups after pulmonary infection with KP B31. *N* = 4–5 mice per group (technical loss during sample preparation). Bar graphs show the quantification of CFU/mg of *Enterobacteriaceae* in feces, and stacked bar graphs represent the relative frequency of *Enterobacteriaceae* isolated from each experimental group. Blue: control diet group. Red: low-fiber diet group. Statistical comparisons between means were performed using unpaired Student's *t*-test. *p < 0.01.

To investigate how a low-fiber diet influences the composition of cultivable *Enterobacteriaceae* species following fecal microbiota transplantation (FMT), we quantified and isolated bacterial strains from MacConkey agar plates before and after infection of germ-free (GF) mice with *K. pneumoniae* B31. All the distinct colonies were isolated, and strain identification was based on by MALDI-ToF analysis (Figure 4A). Before infection, the mice that received FMT from low-fiber diet donors exhibited significantly higher levels of *Enterobacteriaceae*compared to those that received FMT from standard diet donors. In the FMT standard group, the predominant species included *Escherichia coli* (40%), *Enterobacter cloacae* (20%), *Alcaligenes* spp. (20%), *Proteus vulgaris* (10%), and *Providencia rettgeri* (10%). In contrast, the FMT low-fiber group was dominated by *E. coli* (70%), *P. vulgaris* (23%), and *Morganella morganii* (7%) (Figure 4B), indicating reduced species diversity. Following infection with *K. pneumoniae* B31, total *Enterobacteriaceae* levels were comparable between both FMT groups. However, notable shifts in species composition were observed. In the infected FMT standard group, the isolates included *K. pneumoniae* (61%), *E. cloacae* (15%), *P. rettgeri* (8%), *P. vulgaris* (8%), and *E. coli* (8%). In contrast, the FMT low-fiber-infected group was dominated by *K. pneumoniae* (80%), with a lower representation of *P. vulgaris* (10%) and *E. coli* (10%) (Figure 4C). Notably, because the lung bacterial burden after fecal microbiota transplantation was quantified as the total *Enterobacteriaceae* CFU, increased bacterial loads cannot be attributed to *K. pneumoniae B31* alone. Thus, the observed phenotype reflects enhanced pulmonary *Enterobacteriaceae* expansion rather than confirmed pathogen-specific colonization. These results suggest that a low-fiber diet reduces the diversity of cultivable *Enterobacteriaceae* species and favors the dominance of pathogenic strains such as *K. pneumoniae* following infection.

### Short-chain fatty acids counteract gut microbiota shifts induced by low-fiber intake

**Figure 5.**
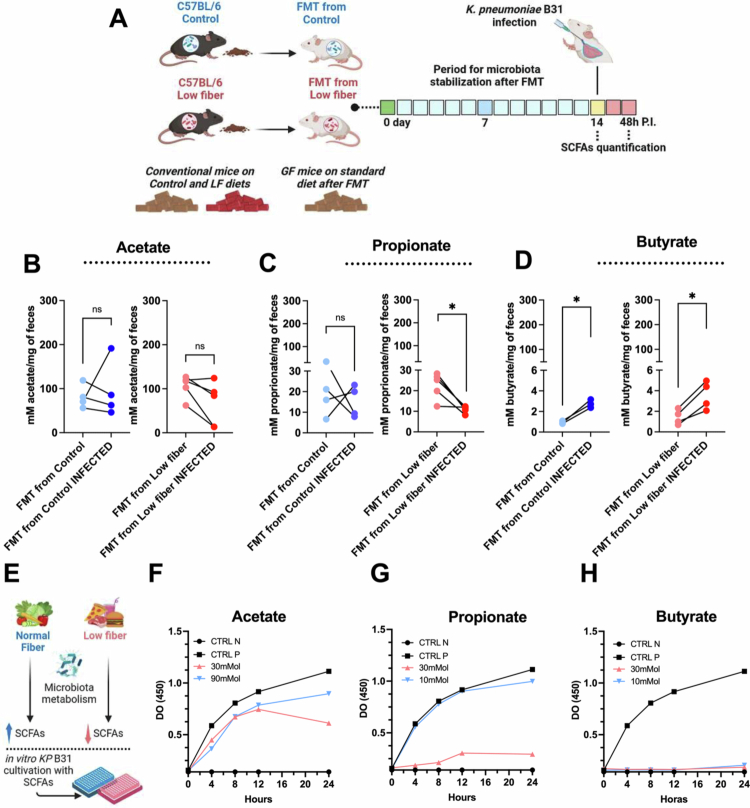
A low-fiber diet alters the metabolic profile of the intestinal microbiota. (A) After FMT from C57BL/6 mice fed with standard diet or low-fiber diet to GF mice, fecal samples from the standard FMT and low-fiber FMT groups were collected and processed before and after KP B31 infection for SCFA quantification. Concentration of (B) acetate, (C) propionate, and (D) butyrate in feces from mice of the FMT from standard and FMT from low-fiber groups, infected and noninfected. (E) Experimental scheme and rationale for the *in vitro* experiment of KP B31 with different SCFAs. *In vitro* growth curves of KP B31 with (F) acetate, (G) propionate, and (H) butyrate. *N* = 4–6 mice per group (variation due to technical issues with the equipment). Light blue: GF mice colonized with microbiota from standard diet-fed mice (FMT from standard), noninfected. Light red: GF mice colonized with microbiota from low-fiber diet-fed mice (FMT from low-fiber), noninfected. (CTRL-N: control medium BHI; CTRL-P: control medium BHI + *K. pneumoniae* B31). Dark blue and dark red: infected animals. Statistical comparisons between means were performed using paired Student's t test. Statistical significance: ns = *p* > 0.05; *p < 0.05.

To explore the role of short-chain fatty acids (SCFAs) in the gut microbiota of GF mice that received fecal samples from the donors under standard versus low-fiber diet, we first quantified the fecal levels of acetate, propionate, and butyrate in the feces of germ-free (GF) mice following fecal microbiota transplantation (FMT) under both noninfected and infected conditions (Figure 5A). No significant differences in acetate levels were observed between the FMT standard and FMT low-fiber groups, either before or after infection (Figure 5B). The propionate levels were similar between the groups before infection (Figure 5C, first graph). In contrast, postinfection analysis revealed a significant decrease in propionate levels, specifically in the FMT low-fiber group (Figure 5C, second graph). Notably, butyrate levels increased significantly in both FMT groups after infection (Figure 5D), indicating that the infectious process may influence SCFA production independently of dietary fiber content. As dietary fiber is a key substrate for microbial SCFA production, its reduction or absence can disrupt microbial metabolic output. Given the known antimicrobial properties of SCFAs, particularly against antimicrobial-resistant *Enterobacteriaceae* [16], we next investigated their direct effects on *K. pneumoniae* B31 growth *in vitro* by treating bacterial cultures with acetate, propionate, or butyrate at varying concentrations (Figure 5E). After 24 h of incubation, we observed a significant reduction in bacterial growth with the lowest dose of acetate (30 mM) (Figure 5F) and with the highest dose of propionate (30 mM) (Figure 5G). Notably, butyrate completely inhibited KP B31 growth at both tested concentrations (10 mM and 30 mM) (Figure 5H). These results suggest that pulmonary infection with KP B31 compromises the ability of the gut microbiota to produce certain SCFAs, particularly propionate, and that fiber deficiency exacerbates this dysfunction.

### Integration of antibiotic resistance and metabolic functions in the gut *Pseudomonadota*

**Figure 6.**
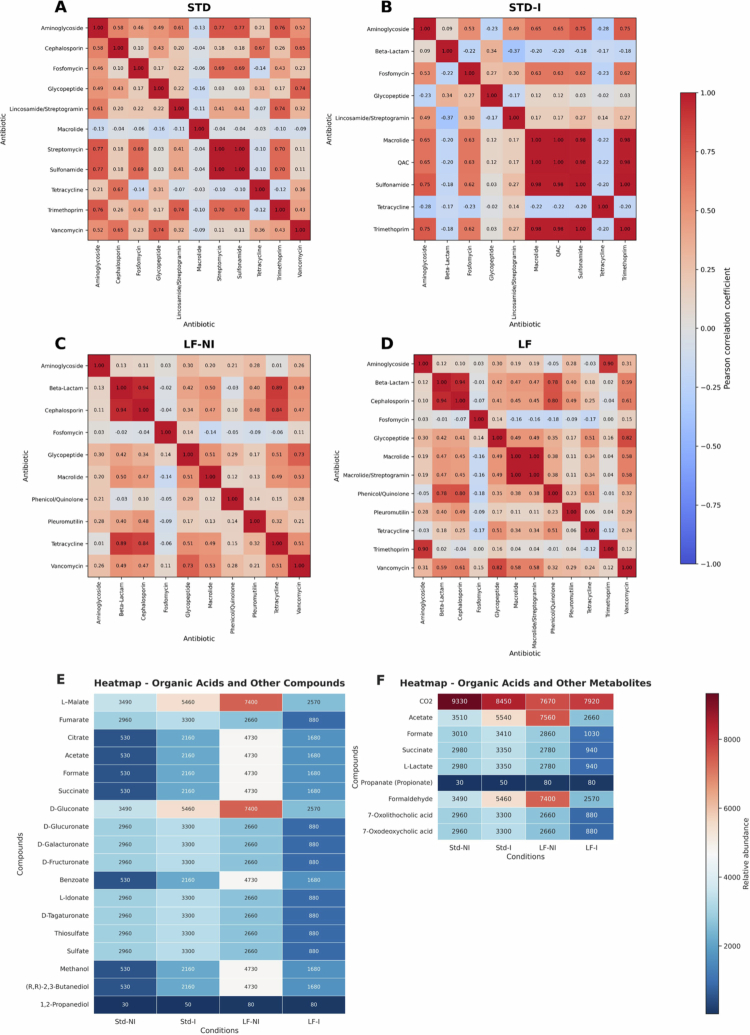
Correlation between antibiotic resistance gene (ARG) classes across experimental groups. Spearman correlation matrices showing pairwise associations between the relative abundances of ARG classes identified in shotgun metagenomic data from four groups: (A) STD (standard diet, no infection, *N* = 4), (B) STD-I (standard diet, with infection, *N* = 3), (C) LF (lower fiber, no infection, *N* = 4), and (D) LF-I (lower fiber, with infection, *N* = 3). The color scale indicates the strength and direction of the correlation coefficients (red, positive; blue, negative; white, near zero). ARGs were annotated using CARD and detected with the tools Abricate and AMRFinder. Strong positive correlations may indicate the co-occurrence or co-selection of resistance mechanisms. (E) Heatmap of organic acids and related compounds. Abundance of bacterial taxa associated with the production or consumption of selected organic acids across the four experimental groups. The values represent the relative abundance of bacteria functionally annotated for each compound. These compounds include intermediates of microbial fermentation and host–microbiota cometabolism, such as D-gluconate, fumarate, succinate, and citrate. A higher abundance (red) indicates greater representation of producer or consumer taxa; a lower abundance (blue) indicates reduced presence. (F) Heatmap of short-chain fatty acids (SCFAs) and other microbiota-associated metabolites. Relative abundance of bacterial taxa linked to the production or consumption of key SCFAs (e.g., acetate, propionate, formate) and host-derived metabolites (e.g., bile acid derivatives) under each experimental condition. Notably, an increase in propionate-associated bacteria was observed in the LF-I group. The heatmap color gradient reflects the abundance of functional microbial groups, from low (blue) to high (red).

To assess patterns of antibiotic resistance within the *Pseudomonadota* phylum, we generated a heatmap based on normalized data to visualize correlations between different antibiotics (Figure 6). Certain antibiotic pairs, such as tetracycline and fosfomycin, displayed weak negative correlations (ranging from −0.14 to −0.23), suggesting distinct co-localization patterns within metabolic pathways (Figure 6A–D). In contrast, a strong positive correlation (*r* = 0.94) was observed between beta-lactam and cephalosporin antibiotics, indicating potential overlap in their associated metabolic pathways (Figure 6C and D). Additionally, beta-lactam and tetracycline showed a high correlation (*r* = 0.89), further suggesting functional linkage or shared resistance mechanisms. These correlations imply that antibiotics with high co-localization may target similar microbial processes, whereas negatively correlated antibiotics might reflect alternative or complementary therapeutic strategies. To further explore functional differences, hierarchical clustering was used to analyze the distribution of cellular metabolic pathway categories across groups. This analysis grouped functionally similar metabolic categories, revealing distinct clustering patterns. In terms of metabolite consumption, infection led to an increase in acetate and [R,R]-2,3-butanediol in both the standard and low-fiber diet groups, with the latter showing a modest 11% increase (Figure 6E). Regarding metabolite production, acetate levels increased after infection in the standard diet group but decreased in the low-fiber diet group when comparing with lower-fiber noninfected diet group (Figure 6F). Together, these findings highlight the complex interplay between antibiotic resistance patterns and microbial metabolic activity in the gut microbiota, particularly within the *Pseudomonadota* phylum. Understanding these associations may aid in the development of more effective, microbiota-informed, and personalized therapeutic strategies for managing bacterial infections.

## Discussion

As AMR continues to escalate globally, there is an urgent need for innovative and complementary strategies to safeguard the efficacy of existing antibiotics. One emerging avenue involves modulating the gut microbiota through dietary interventions approach capable of influencing the resistome (the collective pool of AMR genes) within the host microbiome. In this work, our findings support and expand on these observations, suggesting that short-term dietary imbalances—particularly fiber deprivation—not only reshape gut microbial communities but also may potentiate the spread of resistant bacteria and compromise pulmonary defense mechanisms through systemic microbiota-mediated effects. A key player in this context is the gut–lung axis, a bidirectional communication system that connects the gastrointestinal (GI) and respiratory tracts via blood and lymphatic circulation. This axis enables microbial signals, metabolites, and immune mediators to influence distant organs, including the lungs.^52^ Clinical and experimental evidence increasingly shows that diets deficient in essential nutrients and dietary fibers disrupt host–microbiota interactions along the gut–lung axis. Such disruptions negatively impact microbial composition, immune regulation, and barrier integrity, ultimately increasing susceptibility to respiratory infections and contributing to the development of chronic lung conditions.^53^^,^^54^ Accumulating evidence indicates that dysbiosis induced by low-fiber diets increases susceptibility to pulmonary infections through multiple interconnected mechanisms. These include reduced production of short-chain fatty acids (SCFAs), compromised intestinal barrier integrity, diminished regulatory immune cell populations, and elevated levels of proinflammatory cytokines such as IL-6 and TNF-α.^55-58^ These prior findings are consistent with the results of the present study, in which mice fed a low-fiber diet demonstrated increased susceptibility to KP B31 infection. This was evidenced by greater weight loss, more intense tissue inflammation, and elevated levels of pulmonary neutrophils—hallmarks of a heightened inflammatory response and impaired immune regulation. Because the low-fiber diet was generated by substituting cellulose with sucrose to preserve caloric equivalence, differences between diets extend beyond fiber content alone. Thus, while increased disease severity was observed under low-fiber conditions, contributions from elevated simple carbohydrate intake cannot be excluded, and future sucrose-matched, fiber-controlled diets will be required to isolate fiber-specific effects. During pulmonary infections caused by *K. pneumoniae*, substantial alterations in the composition and function of the intestinal microbiota have been observed. Wu et al. reported reduced microbial diversity and increased colonization by pathobionts such as *Escherichia coli* and *Clostridium methylpentosum*. This dysbiosis was accompanied by a marked reduction in the production of intestinal short-chain fatty acids (SCFAs), particularly following infection.^59^ Moreover, pulmonary infection triggered a strong inflammatory response characterized by elevated levels of proinflammatory cytokines and chemokines—including IL-6, TNF-α, and monocyte chemotactic protein-1 (MCP-1)—in both intestinal and lung tissues, alongside a decline in intestinal barrier integrity. These findings support the notion that respiratory infections can exacerbate diet-induced dysbiosis, reinforcing a bidirectional relationship between the gut and lungs that may amplify both inflammation and susceptibility to antimicrobial-resistant pathogens. Our results of gut microbiota shotgun metagenomic data reveals that both dietary fiber intake and infection status profoundly shape the gut microbial ecosystem at the taxonomic and functional levels. Mice receiving a low-fiber diet showed reduced alpha diversity, which is consistent with prior studies indicating that fiber deprivation leads to microbial extinction and impaired community resilience.^60^^,^^61^ Moreover, principal coordinates analysis (PCoA) of Bray‒Curtis dissimilarities revealed clear separation between groups, indicating significant shifts in community composition (beta diversity). This finding supports evidence from Desai et al. showing that a lack of dietary fiber not only reduces microbial diversity but also disrupts community structure in ways that may favor pathobiont overgrowth.^29^ Within this altered landscape, we observed an increased abundance of opportunistic taxa such as *K. pneumoniae* and *A. baumannii*, particularly under low-fiber and infection conditions. This taxonomic shift was accompanied by functional remodeling, including enrichment of genes involved in virulence, amino acid metabolism, and antibiotic resistance. These patterns mirror findings in human and animal models, where dysbiosis driven by diet or inflammation fosters microbial communities with enhanced metabolic flexibility and pathogenic potential.^62^^,^^63^ Our correlation analyses revealed strong co-occurrence between resistance genes, notably beta-lactam and cephalosporin classes, supporting the idea that environmental stressors such as infection and fiber deprivation may promote co-selection of ARGs.^64^^,^^65^ While dietary fiber supplementation has been proposed to limit ARG proliferation by supporting competitive commensal population,^20^^,^^66^ our findings suggest that infection pressure may override this protective effect. Importantly, the expansion of ampicillin-resistant Enterobacteriaceae was not observed in infected mice fed the standard diet, nor at baseline in either dietary group and was accompanied by a selective increase in *K. pneumoniae* under low-fiber conditions. Together, these observations support an infection-associated expansion of resistant Enterobacteriaceae in the context of fiver deprivation, despite the absence of a time-matched noninfected low-fiber control. Future longitudinal studies including noninfected low-fiber controls will be necessary to disentangle infection-dependent effects from those driven by diet and temporal dynamics.

To further dissect the causal role of the gut microbiota in modulating host susceptibility to respiratory infection, we propose that the increased susceptibility to pulmonary infection observed in fiber-deprived animals is driven primarily by structural and functional changes in the intestinal microbiota. To test this hypothesis, the fecal microbiota from low-fiber-fed mice was transplanted into germ-free recipients, knowing that donor–recipient differences may modulate the magnitude of transferred effects. Remarkably, these recipient mice exhibited a similar vulnerability to *K. pneumoniae* infection as the donor animals, confirming that the heightened susceptibility was microbiota-dependent. It is important to confirm that the microbiota-dependent transfer of disease severity, while acknowledging that additional controls (e.g., heat-inactivated or filtered FMT), would be required to definitively exclude contributions from acellular components. These findings suggest that fiber deficiency alone is sufficient to reshape the gut microbial community in a way that compromises host defense. Interestingly, while fiber deprivation compromises microbiota-mediated protection, our findings are consistent with emerging evidence that prior infections can functionally “train” the microbiota to increase resistance to subsequent pathogenic challenges.^67^ Although we did not directly investigate postinfection microbial memory in this study, our data suggest that fiber deficiency may impair the development of this protective phenotype. This effect may be driven, at least in part, by altered microbial metabolite production—specifically, the modulation of short-chain fatty acids (SCFAs), which are known to play key roles in maintaining immune homeostasis and barrier integrity.^68-70^ Following infection with *K. pneumoniae* B31, we observed a marked reduction in fecal propionate production in germ-free mice receiving FMT from low-fiber diet donors, whereas fecal butyrate levels were increased in both FMT groups after infection. Importantly, fecal SCFAS concentrations represent the net outcome of microbial production, host uptake, and intestinal utilization and may not directly reflect SCFA bioavailability in the mucosal or luminal microenvironments where Enterobacteriaceae expand. Butyrate is normally rapidly consumed by colonocytes as a primary energy source, and increased fecal butyrate has been reported in the context of epithelial dysfunction or altered uptake during inflammation. Thus, the observed increase in fecal butyrate following infection likely reflects altered epithelial handling rather than increased antimicrobial pressure against *K. pneumoniae*. In contrast, propionate production is strongly dependent on fiber-fermenting anaerobic taxa, which are selectively reduced under low-fiber dietary conditions. The reduction in fecal propionate therefore likely reflects a genuine loss of propionate-generating capacity and colonization resistance, which is consistent with the increased dominance of *K. pneumoniae* observed in low-fiber, infected mice. Supporting this interpretation, our *in vitro* assays demonstrated that both propionate and butyrate can directly inhibit *K. pneumoniae* survival in a dose-dependent manner, which is consistent with weak acid–mediated and metabolic stress mechanisms. However, the antimicrobial efficacy of SCFAs *in vivo* is expected to depend on local concentration, pH, and spatial distribution, which may not be fully captured by fecal measurements alone. In this context, a limitation of the present study is that SCFAs were measured in fecal material rather than directly at the mucosal interface or in portal/systemic circulation. Future studies combining spatial metabolite profiling with epithelial uptake measurements will be important to precisely define the bioavailable SCFA concentrations that constrain *K. pneumoniae* expansion during infection.

Although the effects of low-fiber diets on the gut microbiota have been extensively studied,^30^ their impact on immune function in distant organs such as the lungs remains less well understood. Beyond direct antimicrobial effects, SCFAs also exert important immunomodulatory functions.^58^^,^^71^ Neutrophils—critical effectors of the innate immune response—are particularly sensitive to metabolic and inflammatory cues influenced by the gut microbial composition and SCFA levels. SCFAs, particularly acetate, propionate, and butyrate, are major metabolites derived from microbial fermentation of dietary fiber and play a central role in regulating neutrophil activity through GPCRs such as GPR41 and GPR43^58^^,^^71^ demonstrated that the administration of acetate—or oral treatment with *Bifidobacterium longum* 5^1A^, a high-acetate–producing strain—enhanced GPR43 signaling. This activation, primarily in neutrophils and macrophages, was essential for modulating inflammation, boosting phagocytic activity, and promoting bacterial clearance in a pulmonary infection model with *K. pneumoniae*. Thus, reductions in propionate availability under low-fiber conditions may impair both microbiota-mediated colonization resistance and systemic immune responsiveness during lung infection.

These changes are characterized by reduced diversity in antibiotic–microbe associations, shifts in metabolic pathway usage, and selective disruption of short-chain fatty acid (SCFA) production—most notably propionate—under infection conditions. Together, these alterations suggest that fiber deficiency not only disturbs microbial metabolic balance but also creates conditions that may promote the emergence and persistence of antimicrobial-resistant strains, thereby increasing the risk of resistance gene dissemination and undermining antibiotic efficacy. Given the growing global threat of AMR, there is an urgent need for innovative, complementary strategies to support current antibiotic treatments. Our findings highlight the potential of dietary interventions—specifically targeting gut microbiota modulation through increased fiber intake—as a scalable, nonpharmacological approach to influence the resistome and enhance resistance management efforts.

## Supplementary Material

Supplementary materials 23012026.docx Supplementary materials 23012026.docx

## Data Availability

The metagenomic libraries produced in this work were deposited in the NCBI SRA database and available under project number PRJNA1265282.
