# Supplementary material for: Fiber-deprived diet weakens lung defense against antimicrobial-resistant Klebsiella pneumoniae and facilitates resistance phenotype in the gut microbiota
Source: Gut Microbes Rep. 2026 Feb 8;3(1):2625617. doi: 10.1080/29933935.2026.2625617 (PMC12938878; doi:10.1080/29933935.2026.2625617)
Supplement: Supplementary materials 23012026.docx [file KGMR_A_2625617_SM1009.docx]

**Supplementary Materials**

**Fiber-deprived diet weakens lung defense against antimicrobial-resistant *Klebsiella pneumoniae* and facilitates resistance phenotype in the gut microbiota**

Mayra Fernanda Ricci^1*#^, Clenio Silva Cruz^1*^, Viviani Mendes de Almeida^1^, Mirna d’ Auriol^2^, Victor M. Rocha^1^, Elayne C. Machado^1^, Bruno Gallotti^1^, Isabela Garbazza^1^, Ana Maria Caetano Faria^3^, Geovanni Dantas Cassali^4^, Cristiana C. Garcia^5,6^, Leiliane Coelho André^2^, Flaviano S. Martins^7^, Vinícius Abreu^8^, Sintia Almeida^9^ and Angélica Vieira^1,10#^

**Supplementary Figure S1. Standardization of the Low-Fiber Diet model.**

**(A)** Percentage composition (%) of macro- and micronutrients in the Standard Diet and Low-Fiber Diet. **(B)** Schematic representation of the feeding schedule for conventional C57BL/6 mice with respective diets. **(C)** Body weight evolution of C57BL/6 mice fed a Standard Diet or Low-Fiber Diet over 21 days. **(D)** Intestinal transit time assessment in C57BL/6 mice subjected to Standard and Low-Fiber Diets. Blue: Standard Diet group. Red: Low-Fiber Diet group. Statistical comparisons between means were performed using unpaired Student's t-test. ns = p > 0.05.

**
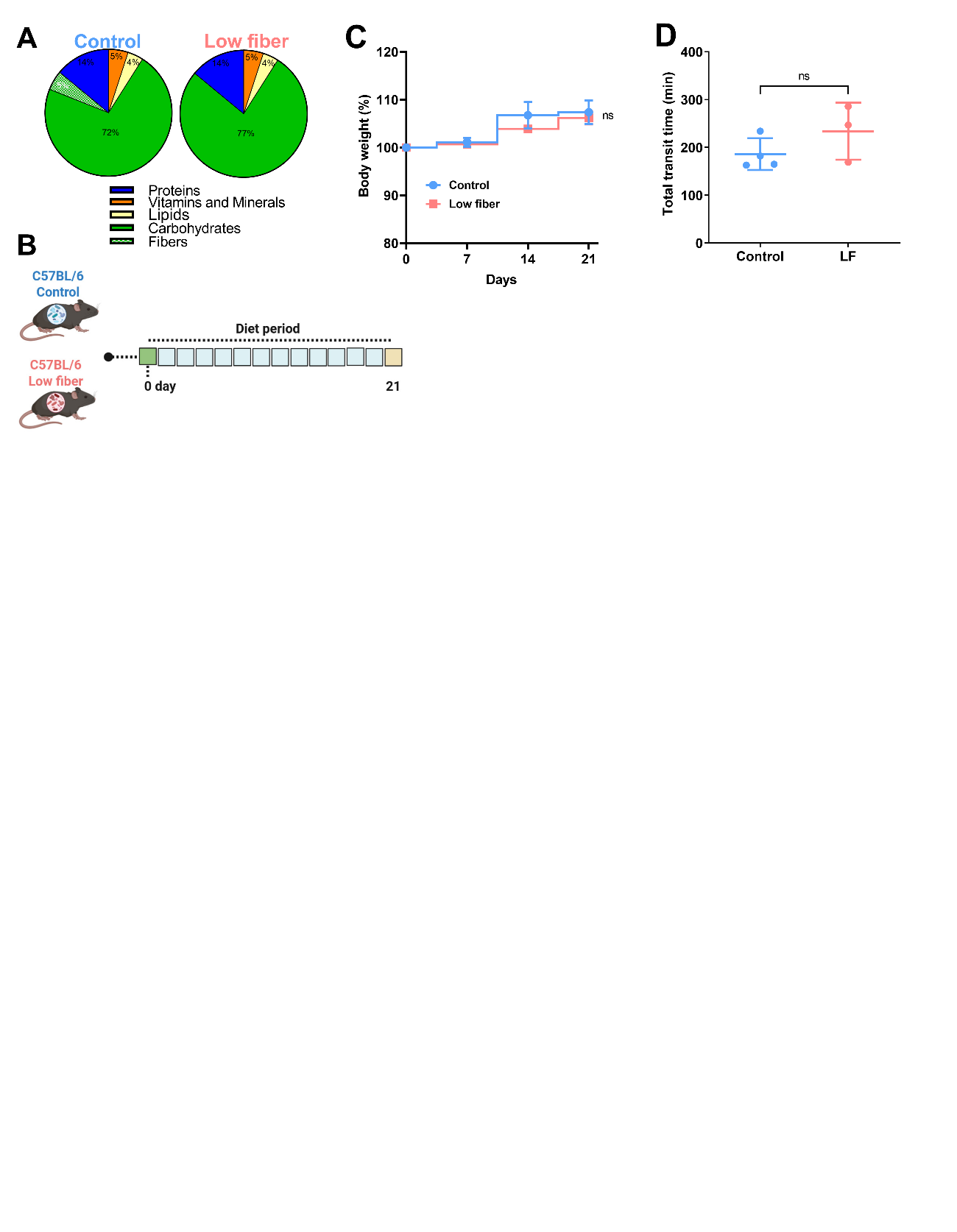
**

**Supplementary Table S1.** Diets used in this study

|  | Quantity (g/kg) | |
| --- | --- | --- |
| Ingredients | Control (AIN-93M) | Low-Fiber |
| Butylated hydroxytoluene | 0.008 | 0.008 |
| Choline bitartrate | 2.5 | 2.5 |
| Cystine | 1.8 | 1.8 |
| Vitamin mix | 10.0 | 10.0 |
| Mineral mix | 35.0 | 35.0 |
| Cellulose | 50.0 | 0 |
| Soya oil | 40.0 | 40.0 |
| Sucrose | 100.0 | 100.0 |
| Maltodextrin | 155.0 | 205.0 |
| Casein | 140.0 | 140.0 |
| Maize starch | 465.7 | 465.7 |
| Distilled water | 300.0 | 300.0 |
| Pectin | 0 | 0 |

**Supplementary Table S2.** **|** **Dunn's Post-hoc Test for Shannon Diversity.**

This table presents the results of Dunn's post-hoc test for pairwise comparisons of Shannon diversity for the comparisons between LF-I, LF-NI, Std-I, and Std-NI. Adjusted p-values account for multiple comparison corrections, with values below 0.05 considered statistically significant.

| **Comparison** | **Z** | **P.unadj** | **P.adj** |
| --- | --- | --- | --- |
| LF-I - LF-NI | 0.3651484 | 0.71500065 | 0.71500065 |
| LF-I - Std-I | 0.7807201 | 0.43496716 | 0.65245074 |
| LF-NI - Std-I | 0.4694765 | 0.63872909 | 0.76647491 |
| LF-I - Std-NI | 2.4777925 | 0.01321980 | 0.07931880 |
| LF-NI - Std-NI | 2.2819165 | 0.02249427 | 0.06748281 |
| Std-I - Std-NI | 1.6431677 | 0.10034825 | 0.20069649 |

**Supplementary Table S3.**

Pairwise comparison of beta diversity among experimental conditions based on Bray–Curtis dissimilarity. PERMANOVA tests were performed using 999 permutations to assess differences in microbial community structure across dietary (Standard vs. LowFiber) and infection (Control vs. Infected) conditions. Strongest differences were observed between dietary groups (LowFiber vs. Standard: p.adjusted = 0.001), while infection alone did not explain significant variation in community structure. Pairwise differences between some condition-level combinations (e.g., LF-I vs. Std-NI) were significant before correction but lost significance after multiple testing adjustment.

| **pairs** | **Df** | **SumOfSqs** | **F.Model** | **R2** | p.value | p.adjusted |
| --- | --- | --- | --- | --- | --- | --- |
| LF-I vs LF-NI | 1 | 0.04444327 | 2.082362 | 0.2940209 | 0.125 | 0.750 |
| LF-I vs Std-NI | 1 | 0.66873851 | 17.674203 | 0.7794851 | 0.026* | 0.156 |
| LF-I vs Std-I | 1 | 0.59280861 | 15.432413 | 0.7941583 | 0.100 | 0.600 |
| LF-NI vs Std-NI | 1 | 0.74931197 | 18.143449 | 0.7514854 | 0.024* | 0.144 |
| LF-NI vs Std-I | 1 | 0.69907337 | 16.467086 | 0.7670853 | 0.041* | 0.246 |
| Std-NI vs STD-I | 1 | 0.12502979 | 2.121053 | 0.2978567 | 0.183 | 1.000 |
| LowFiber vs Standard | 1 | 1.281409 | 26.93346 | 0.6917819 | 0.001** | 0.001** |
| Infected vs Control | 1 | 0.108761 | 0.7485403 | 0.05871576 | 0.579 | 0.579 |

Signif. codes: 0 ‘***’ 0.001 ‘**’ 0.01 ‘*’ 0.05 ‘.’ 0.1 ‘ ’ 1
